# Effect of Smoking on Seminal Plasma Vitamin B_6_ in Fertile and Subfertile Men

**DOI:** 10.1155/2024/8159993

**Published:** 2024-09-03

**Authors:** Shefa' Muneer Aljabali, Saleem Ali Banihani

**Affiliations:** Department of Medical Laboratory Sciences Faculty of Applied Medical Sciences Jordan University of Science and Technology, P.O. Box 3030, Irbid 22110, Jordan

## Abstract

Despite extensive documentation of the negative consequences of smoking on a wide range of diseases and disorders, the direct effect of smoking on seminal plasma vitamin B_6_ (vitB_6_) concentration is not addressed yet. Here, we attempted to examine the influence of smoking on the levels of seminal plasma vitB_6_ in fertile and subfertile males. One hundred and ninety-five participants were categorized into two groups: fertile men (smokers (*n* = 32), nonsmokers (*n* = 43)) and subfertile men (smokers (*n* = 65), nonsmokers (*n* = 55)). According to the World Health Organization criteria, conventional semen analysis was performed for all subjects involved in the study. The concentration of vitB_6_ in semen samples was measured by high-performance liquid chromatography tandem-mass spectrometry. There was no significant difference in the mean seminal plasma concentrations of vitB_6_ in smokers versus nonsmokers in both fertile (*p*=0.5096) and subfertile (*p*=0.5224) groups. Besides, no significant correlations were observed between seminal plasma concentration of vitB_6_, duration of smoking, and men's age in all tested groups. In conclusion, these findings elucidate that smoking has no significant impact on the levels of seminal plasma vitB_6_ in both fertile and subfertile men.

## 1. Introduction

Vitamin B_6_ (vitB_6_) is a vitally important factor in various physiological, developmental, and metabolic processes [1, 2]. It was shown that pyridoxal-5′-phosphate, the biologically active form of vitB_6_, facilitates diverse biochemical reactions involved in the metabolism of fatty acids, amino acids, and carbohydrates [1]. In a broad array of research studies, the effect of vitB_6_ on human's health was extensively demonstrated. For instance, it was noted that vitB_6_ intake and supplementation are significantly associated with minimized risk of diabetes [3, 4], cancer [5], cardiovascular diseases [6–8], sarcopenia [9], and cognitive-related diseases [10, 11].

Yet, there have been only limited studies on the influence of vitB_6_ in boosting human semen parameters and hence the fertilizing capacity [12]. So far, it has been documented that vitB_6_ is existing in human semen, but investigations in this research approach failed to demonstrate its direct effect on semen quality and ability of fertilization, though there are intriguing inferences that depressed levels of vitB_6_ in semen are linked to poor semen quality parameters [12]. Our study in 2020, albeit partially, has confirmed these indirect conclusions which demonstrated that asthenozoospermic semen samples significantly contain less content of vitB_6_ when compared to normozoospermic samples [13]. The findings from this research provide additional support to consider that normal amounts of vitB_6_ in semen are highly recommended to maintain normal semen quality characteristics. Consequently, potential factors that might affect the levels of vitB_6_ in semen should be assessed and controlled. One of such important factors is smoking since it is known to be highly common in men of reproductive age [14].

According to the World Health Organization, tobacco smoking is considered as epidemic [15]. Young adult men during their reproductive period were found to have the highest prevalence of smoking worldwide [14]. Numerous studies have reported that smoking exerts various detrimental effects on humans' health [16, 17]. For example, smoking was found to increase the risk of cardiovascular diseases, lung diseases, cancers, and diabetes [18–20]. Among other consequences, there is evidence of increased inflammation, oxidative stress, and decreased levels of antioxidant vitamins [21, 22]. Ascorbic acid and *β*-carotene are antioxidant micronutrients known to be greatly affected by smoking status [23–28]. However, the evidence for a link between tobacco smoking and circulating concentrations of vitB_6_ is equivocal. On the one hand, some studies revealed that there is no statistically significant difference regarding plasma vitB_6_ levels in smokers versus nonsmokers [29, 30]. On the other hand, other studies revealed that serum vitB_6_ levels were considerably lower in smokers when compared to nonsmokers [31, 32].

Consequently, even though there are several published articles that are documenting the impact of smoking on the levels of vitB_6_ in blood, its effect on the levels of vitB_6_ in semen ejaculates is not addressed yet. Therefore, we asked whether smoking has a direct influence on the levels of vitB_6_ in the human ejaculated semen. Here, we hypothesized that the levels of seminal plasma vitB_6_ are negatively affected by tobacco smoking. To accurately test this hypothesis, we have used the high-performance liquid chromatography tandem-mass spectrometry (HPLC-MS/MS) as a highly robust and reliable analytical method.

## 2. Materials and Methods

### 2.1. Study Population and Specimen Collection

One hundred ninety-five male participants, aged 19 to 47 years, were randomly recruited from the In Vitro Fertilization (IVF) Center at King Abdullah University Hospital (KAUH) and Al-Qudah Private Laboratories in Irbid, Jordan. Study subjects were categorized into two groups: fertile men (smokers (*n* = 32), nonsmokers (*n* = 43)) and subfertile men (smokers (*n* = 65), nonsmokers (*n* = 55)). Semen ejaculates were collected via masturbation after 3–7 days of sexual abstinence.

Prior to sample collection, all study subjects filled out a general questionnaire in both locations. Smoking status, duration of smoking, age, diseases, family history, and the use of medication or vitamin supplements were data extracted from the questionnaire. The cases with varicoceles, chronic diseases, family history with infertility, and cases who are on vitamin supplementation or on medication were excluded from the study.

### 2.2. Ethical Approval

This research study was approved by the Research Ethics Committee (#: 193–2018) at Jordan University of Science and Technology (JUST). The study's aims and analysis were clearly explained to the recruited men by qualified researchers at Al-Qudah Private Laboratory and the IVF Center in the JUST University Hospital, Irbid province. Prior to participation, each recruited male, at both locations, voluntarily signed an informed consent form.

### 2.3. Semen Analysis and Storage

After liquefaction, semen parameters were immediately analyzed according to the World Health Organization guidelines 2010 (normal criteria for fertile men: semen volume ≥1.5 mL, progressive motility of sperm ≥32%, sperm count ≥15 million/mL, normal sperm morphology ≥4.0%) [33]. Males were considered subfertile when sperm count is less than 15 million/mL and/or sperm progressive motility is less than 32% [33]. For better accuracy, at least two separate analyses were performed on semen specimens. Afterwards, samples were centrifuged for 10 minutes at 2500 x g and the seminal plasma (supernatant) was isolated and stored without any additives at −20°C for HPLC-MS/MS analysis.

### 2.4. Measurement of Sperm Motility and Concentration

Sperm motility and concentration were evaluated using a phase contrast microscope and Makler counting chamber (Irvine Sci., USA). After gentle mixing, each semen sample (∼10 µL) was assessed for sperm motility and concentration in a period of 10–15 minutes after sample collection. Total and progressive motility were calculated in percentages, whereas sperm concentrations were measured in 10^6^/mL of semen. Despite the speed of spermatozoa, circularly or linearly active motile sperm were considered progressive. To achieve accurate results, a total of 200 spermatozoa were estimated in each replicate to determine the percentage of both total and progressive motility. Furthermore, to minimize the assessment's bias, all scanned sperm fields were chosen at random. Positive error in the results was avoided by counting the sperm cells quickly [34]. The sperm motility and concentration measurements were performed by highly skilled andrologists who achieve within-individual variation of less than ±5%.

### 2.5. Seminal Plasma Sample Preparation

Seminal plasma specimens were thawed at room temperature and mixed on a vortex mixer. Protein precipitation was achieved by mixing 250 *μ*L of seminal plasma with 3 mL of chloroform and 250 *μ*L of ethanol. After that, the mixture was completely vortexed for 30 s and then centrifuged at 2,500 g for 10 min. Supernatants were transferred into a clean microfuge tube and then vaporized completely in a N_2_ evaporator. Next, 750 *μ*L of methanol was added to the pellet and the final suspension was subsequently placed into an autosampler vial for HPLC-MS/MS.

### 2.6. Measuring VitB_6_ Levels by HPLC-MS/MS

The concentrations of vitB_6_ in seminal plasma were determined using the 30AD SHIMADZU Binary HPLC system coupled with an 8,030 ESI-MS/MS system (SHIMADZU Corp.) [35]. In summary, the separation analysis was mainly carried out by injecting 10 *μ*L sample volume into the revered phase HPLC column (Zorbax Eclipse Plus C18, 1.8 *μ*m particle size, 30 × 2.1 mm) (SHIMADZU Corp.). The mobile phase was made up of 2% methanol, 4.8 g/L ammonium carbonate, and 98% water. The analytes of interest were separated by an isocratic elution at a flow rate of 0.4 mL/min. The quantitative determination was achieved on the tandem mass spectrometer with the following conditions: gas flow = 10 L/min, sheet gas flow = 11 L/min, gas temperature = 200°C, sheet gas heater = 350°C, charging voltage = 50 V, capillary voltage = 3500 V, and nebulizer pressure = 40 psi. All data were obtained using positive mode electrospray ionization (ESI) and all measurements were achieved in duplicate with sensitivity of 0.01 *μ*g L^−1^.

LC-MS/MS is a highly accurate and precise method for quantifying vitB_6_, particularly pyridoxal-5′-phosphate (PLP), in biological samples. The method demonstrates excellent intra-day and inter-day precision, with coefficients of variation typically below 10.4% [35]. The accuracy is also high, with recovery rate of approximately 100% [35]. Additionally, the method offers a very low limit of detection, around 25.9 nmol/L, making it suitable for detecting trace amounts of PLP [35]. The total imprecision for PLP ranges from 30 to 130 nmol/L [35].

### 2.7. Statistical Analysis

Data were represented as mean ± standard error of the mean. The statistical appraisals and analysis were carried out using GraphPad Prism 5.01 Computer Software (GraphPad Software Inc.). Student's *t* test was performed to examine the differences in the mean seminal plasma vitB_6_ levels in smokers versus nonsmokers in both fertile and subfertile groups. Pearson's correlation coefficient was used to compute the correlations between seminal plasma vitB_6_ concentration, duration of smoking, and men's age in the tested groups. Results were considered statistically significant at “*p*” value <0.05.

## 3. Results

In this work, semen samples were obtained randomly from different male subjects (*n* = 195).  Table 1 shows the preliminary results of conventional semen parameters and seminal plasma vitB_6_ levels for the recruited samples for fertile (*n* = 75) and subfertile (*n* = 120) men. Results of semen volumes, sperm counts, sperm motilities (total and progressive), and seminal plasma vitB_6_ concentrations were expressed as mean ± standard error of the mean. Males were considered subfertile when sperm count is less than 15 million/mL and/or sperm progressive motility is less than 32% [33].

**Figure 1:**
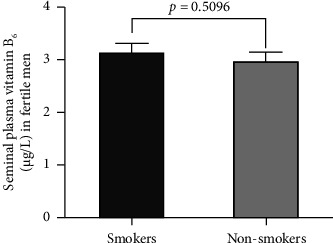
Concentrations of seminal plasma vitB6 in fertile smokers (*n* = 32) compared to fertile nonsmokers (*n* = 43). Data are represented as mean ± standard error of the mean.

Figure 1 exhibits the difference in mean seminal plasma vitB_6_ levels—as evaluated by HPLC-MS/MS—in smokers (*n* = 32) versus nonsmokers (*n* = 43) in the fertile group. As displayed in the figure, there is no statistically significant difference (*p*=0.5096) in the concentrations of seminal plasma vitB_6_ in smokers when compared to nonsmokers in this group.

**Figure 2:**
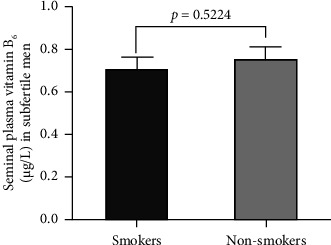
Concentrations of seminal plasma vitB6 in subfertile smokers (*n* = 65) compared to subfertile nonsmokers (*n* = 55). Data are represented as mean ± standard error of the mean.

The comparison in mean seminal plasma vitB_6_ concentrations in smokers (*n* = 65) versus nonsmokers (*n* = 55) in the subfertile group is illustrated in Figure 2. Statistically, there is no significant difference in the mean seminal plasma vitB_6_ levels (*p*=0.5224) in smokers versus nonsmokers in this group.

**Figure 3:**
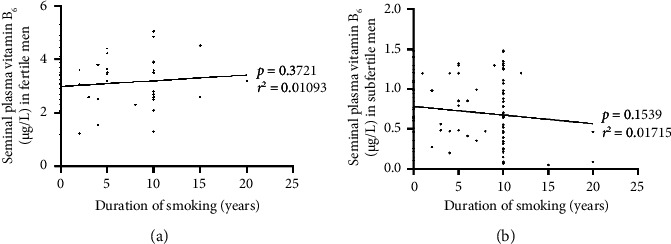
Correlations between seminal plasma vitB6 concentrations in fertile men (a) (*n* = 75) and subfertile men (b) (*n* = 120) and duration of smoking.

The correlations between vitB_6_ levels in seminal plasma and duration of smoking in both fertile (Figure 3(a), *n* = 75) and subfertile (Figure 3(b), *n* = 120) groups are demonstrated in Figure 3. As elucidated in the figure, the correlations between seminal plasma vitB_6_ levels and duration of smoking in fertile men (*p*=0.7321, *r*^2^ = 0.01093) and subfertile men (*p*=0.1539, *r*^2^ = 0.01715) were not statistically significant.

**Figure 4:**
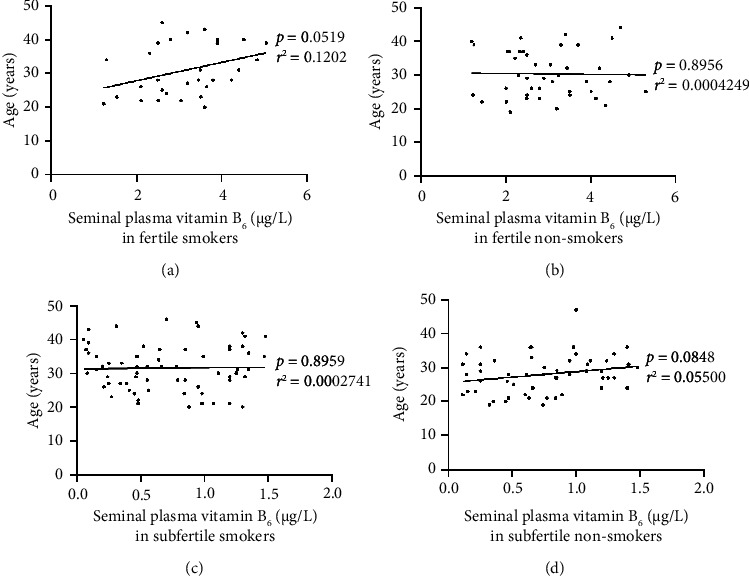
Correlations between seminal plasma vitB6 concentrations in fertile men ((a) smokers (*n* = 32); (b) nonsmokers (*n* = 43)) and infertile men ((c) smokers (*n* = 65); (d) nonsmokers (*n* = 55)) versus men's age.

Figure 4 represents the correlations between vitB_6_ concentrations in seminal plasma and men's age (19–47 years) in fertile smokers (Figure 4(a)), fertile nonsmokers (Figure 4(b)), subfertile smokers (Figure 4(c)), and subfertile nonsmokers (Figure 4(d)). As depicted in the figure, even though seminal plasma vitB_6_ concentrations and men's age were positively correlated in both fertile smokers (*p* = 0.0519, *r*^2^ = 0.1202) and subfertile nonsmokers (*p*=0.0848, *r*^2^ = 0.05500), these correlations were not statistically significant. In fertile nonsmokers (*p*=0.8956, *r*^2^ = 0.0004249) and subfertile smokers (*p*=0.8959, *r*^2^ = 0.0002741), also, this correlation was seen to be not statistically significant.

## 4. Discussion

**Figure 5:**
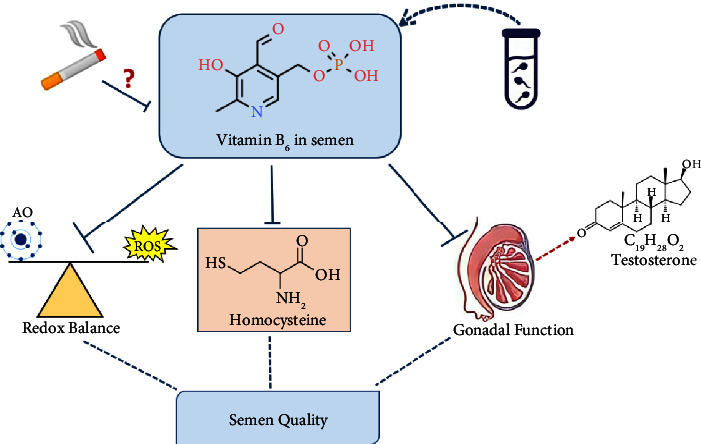
Possible mechanistic routes for the impact of vitB_6_ on semen quality in men.

In 2017, our systematic review has strongly suggested that vitB_6_ deficiency might have a negative impact on sperm parameters [12]. This suggestion was drawn based on intriguing findings from different previous research investigations. In fact, several studies have established that vitB_6_ concentration is positively correlated with the levels of sex hormones, mainly testosterone [36–38] (Figure 5). This finding endorses that vitB_6_ is essential in maintaining normal gonadal function. Additionally, it is postulated that vitB_6_ might protect sperm cells from possible oxidative injury since it acts as a potent antioxidant [39, 40] and maintains the normal levels of reduced glutathione in blood [41, 42]. Moreover, due to the important role of vitB_6_ in converting homocysteine, a toxic byproduct, into cysteine amino acid, it is speculated that vitB_6_ supplementation might reduce the possible chemical toxicity to sperm induced by hyperhomocysteinemia [43, 44]. In 2020, our recent study demonstrated that asthenozoospermic semen samples significantly contain less content of vitB_6_ than normozoospermic samples [13]. The findings from this research provide additional support to consider that normal amounts of vitB_6_ in semen are vital and highly recommended to maintain normal sperm characteristics [13]. Subsequently, investigating potential factors, including smoking and men's age, that might affect the level of vitB_6_ in semen should be deliberately assessed and controlled.

Here, in this work, we attempted to identify the direct effect of smoking on seminal plasma vitB_6_ concentrations in men. We hypothesized that smoking has a negative impact on the seminal plasma vitB_6_ levels in fertile and subfertile men. However, the findings were not in agreement with our central hypothesis. Our data analysis has revealed that there is no difference in seminal plasma vitB_6_ levels between smokers and nonsmokers in either fertile or subfertile men. Besides, it has been shown that the correlations between seminal plasma vitB_6_ concentrations and duration of smoking in both studied groups are not statistically significant.

In 1986, Ritchie and Singkamani examined the blood plasma vitB_6_ concentrations in nonsmoking and smoking females with premenstrual syndrome (PMS) and compared them with the blood plasma vitB_6_ values in nonsmoking and smoking females who did not have PMS. In either group, there was no difference in plasma vitB_6_ levels between nonsmokers and smokers [29]. Also, a study published by Giraud et al. revealed that there were no changes in the plasma vitB_6_ values of young males who smoked, chewed, or never used tobacco [30]. A later research has provided, albeit partially, similar findings when vitamin B_6_, B_9_, and B_12_ were measured in 285 pregnant women at 18 and 30 weeks gestation [45]. Specifically, it was found that pregnant smokers have lower vitB_6_ values in plasma when compared to pregnant nonsmokers at 18 weeks gestation, whereas no such disparity was detected at 30 weeks gestation. Moreover, no significant association was seen between levels of vitB_6_ in plasma and thiocyanate, a biomarker of smoking, in pregnant smoker group at both 18 and 30 weeks gestation [45]. These findings are coherent with our findings.

On the contrary, our findings are not in accordance with some results in other studies. Vermaak et al. reported that serum PLP levels were lower in smokers when compared to ex-smokers and nonsmokers, although PLP levels in erythrocytes did not significantly differ between the tested groups. The authors felt that the depressed levels of PLP in serum are uncertain and measurement of PLP in erythrocytes is more accurate since the PLP is an intracellular coenzyme and more liable in blood plasma [31]. Another study reported similar results in which tobacco users and nonusers had different levels of certain B_6_ vitamers (PLP and pyridoxine 5′-phosphate) in their plasma but not in their erythrocytes [46]. In 1986, a research study compared the levels of plasma PLP in tobacco smoking men to those of nonsmoking controls. The authors observed that tobacco smoking men were found to have lower concentrations of plasma PLP when compared to nonsmoking men [32]. The discrepancy between our study and these studies might be ascribed to variations in the sample type and nature of the tested subjects, as well as detection methods. Here, in the present study, we examined specifically smoking/nonsmoking fertile and subfertile men to evaluate levels of vitB_6_ by HPLC-MS/MS in semen specimens while these previous studies examined the levels of blood plasma vitB_6_ in completely different populations and employed different methodologies.

Smoking accelerates the production of reactive oxygen species, leading to cellular oxidative stress. This cellular imbalance can deplete antioxidant reserves, including vitB_6_, and cause damage to cell components. However, the human body has adaptive mechanisms to maintain essential nutrient levels. VitB_6_, in its active form, PLP, is vital for various physiological functions, including neurotransmitter synthesis, amino acid metabolism, and immune function [47]. The body's ability to sustain PLP levels under oxidative stress may be attributed to increased dietary intake, enhanced absorption, or more efficient recycling and utilization of the vitamin [48]. Moreover, smokers often exhibit altered antioxidant defense systems, which may include compensatory mechanisms to maintain vitB_6_ levels. For example, the liver, which plays a crucial role in metabolizing and storing vitamins, might compensate by more tightly regulating vitB_6_ metabolism in smokers [49]. This could explain the lack of significant differences observed between the two groups in the study.

Moreover, in this study, although we recognized that seminal plasma vitB_6_ concentrations and men's age were positively associated in fertile smokers and subfertile nonsmokers, these associations were not statistically significant. Also, this correlation was seen to be not statistically significant in fertile nonsmokers and subfertile smokers. In 1976, Rose et al. discovered an inverse relationship between blood plasma vitB_6_ levels and male age [50]. In fact, our correlation focused primarily on fertile and subfertile men who smoked or never smoked, aged from 19 to 47 years, whereas Rose et al. included community-dwelling men, aged from 18 to 90 years. Also, here, we evaluated specifically vitB_6_ in seminal plasma using HPLC-MS/MS, whereas Rose et al. evaluated vitB_6_ in blood plasma using simple enzymic assay. Such age, population, and methodological differences might explain the observed contradiction between the two studies. According to such specific investigation, levels of vitB_6_ in semen seem not to be altered by factors like men's age or smoking status, which may suggest that depressed levels of vitB_6_ in semen can be mainly attributed to nutritional factors. However, this suggestion requires more research studies to be confirmed.

Diet and lifestyle factors markedly influence vitB_6_ status, with recent research highlighting their complex roles. A diet rich in vitB_6_, found in foods like fish, poultry, potatoes, and bananas, is vital for maintaining optimal concentrations of this vitamin [51]. Conversely, lifestyle factors such as alcohol consumption and chronic stress, in addition to smoking, can negatively affect vitB_6_ metabolism [52]. For example, alcohol impairs the conversion of pyridoxine to PLP, leading to reduced levels even in subjects with sufficient dietary intake [52]. Furthermore, chronic stress has been associated with an increased demand for B vitamins, including vitB_6_, due to their roles in neurotransmitter synthesis and adrenal function [52]. This evidence underscores the importance of a balanced diet and healthy lifestyle choices in supporting adequate levels of vitB_6_, particularly for those with high-risk behaviors.

In this study, cases involving varicoceles, chronic diseases, a family history of infertility, and individuals on B vitamin supplementation or medication were excluded from all groups. However, controlling the diet of the recruited participants remains a limitation due to the random selection process and the inherent variability of dietary factors. Additionally, other limitations arose from confounding variables such as socioeconomic and environmental factors (e.g., pollution and occupational exposure), which, if controlled, could further strengthen the study's findings. Therefore, an interventional study in this research setting is deemed essential to validate and confirm the results of this study.

In conclusion, there is no change in the level of seminal plasma vitB_6_ between smokers and nonsmokers in both fertile and subfertile groups. Also, the seminal plasma vitB_6_ concentrations are not correlated with either men's age or smoking duration in the studied groups. Collectively, these results manifest that smoking has no significant influence on seminal plasma vitB_6_ in fertile or subfertile men.

## Figures and Tables

**Table 1:** The conventional semen parameters and seminal plasma vitB6 concentrations for recruited semen samples for both fertile and subfertile men.

Measured parameter	Results	
	Semen samples for fertile men (*n* = 75)	Semen samples for subfertile men (*n* = 120)
Total semen volume (mL)	3.745 ± 0.1987	3.143 ± 0.1301
Sperm count (10^6^/mL)	55.61 ± 2.307	26.49 ± 1.229
Total sperm motility (%)	67.07 ± 1.274	35.93 ± 1.028
Progressive motility of spermatozoa (%)	47.87 ± 1.027	19.08 ± 0.6926
Semen viscosity (1, normal; 2, abnormal)	“1,” normal; for all collected samples	“1,” normal; for all collected samples
Normal forms of spermatozoa (%)	≥4%; for all collected samples	≥4%; for all collected samples
Seminal plasma vitB_6_ concentration (*μ*g/L)	3.058 ± 0.1153	0.7298 ± 0.03820

The data are represented as mean ± standard error of the mean.

## Data Availability

The data that establish the results of this work are only available upon reasonable request.
